# Health Tract, No. 241

**Published:** 1866-06

**Authors:** 


HEALTH TRACT, No. 241.

### FEAR OF CHOLERA.

There is something in the air we breathe, which makes the system susceptible of cholera. But as powder is susceptible of explosion, but cannot explode unless a spark of fire is applied, which spark is the immediate cause of the explosion, so a cholera atmosphere will not cause cholera in any case, unless an immediate cause is applied, capable of bringing out the actual disease, and which would not have been manifested without such application.

H. J. Phillips, Surgeon in the U. S. Army, relates in the Medical and Surgical Journal for November, 1865, that while he was stationed at the Military Hospital, at Valetta, on the Island of Malta, in 1855, a sentry was placed on duty, in the passage of a cholera ward, unexpectedly. As soon as he learned that some cholera patients had been brought into the building, he became so alarmed that he was obliged to be relieved, nothing that could be said had any effect in quieting his fears, and he died of the disease within a few hours.

An engineer who had seen persons in the stage of collapse, when the skin is almost black, or of a dark leaden hue, bad, in working among the machinery in a dark room, unconsciously discolored his hands and arms; on coming to the light he immediately perceived the discoloration, and immagining that it was the cholera, he died the same day.

It has been stated that permission was given by a despotic government to take ten men condemned to death; five were put in beds where cholera, patients had just died, and five in fresh beds; they were informed the reverse of the facts; the next day the men who had slept in fresh beds were attacked with cholera, and those who occupied the other beds escaped any attack. A wheeling Editor, with a view to testing the fact of the communicability of cholera, went on board a steamboat in the evening, wrapped himself in the bedding, in which a man had just died of cholera, and remained in it until next morning and was not attacked. These facts show that fear, when cholera is prevailing can excite an attack, because fear relaxes the whole nervous system, it has a most prostrating effect. Cholera is a universal relaxation. Cases are very common where under sudden depressing excitement diarrhœa takes place, and cholera is only an exaggerated diarrhœa. These things prove clearly that when cholera is prevailing in a community, the timid should be promptly removed to some locality where it is not prevailing, and this will be their safety. It does no good and is rather tantalizing to say to such, there is no danger; they cannot help their fears, and as long as these exist, there is very iminent danger, and the sooner they are removed to some exempted place and thus regain their equanimity the safer and better. Let the removal be made cheerfully, without opposition, without impatience or moodiness, and the results will be that much more gratifying. The lesson of the article is, whatever depresses the mind, whatever unpleasantly affects it, can excite the disease within the hour, while a calm courage and self-possession, can defy it.

